# The King at the Orthopædic Hospital

**Published:** 1909-07-31

**Authors:** 


### THE KING AT THE ORTHOPÆDIC HOSPITAL.

His Majesty the King, accompanied by the Queen and Princess Victoria, opened the new in-patient department of the Royal National Orthopædic Hospital, Great Portland Street, W. on July 23. The Royal party drove in semi-state from Buckingham Palace, and were cheered enthusiastically along the route. Their Majesties were received at the main entrance of the hospital by the Duke of Marlborough, president, Lord Denbigh, chairman, and the principal members of the governing body and of the executive and medical staff. The Royal visitors were then escorted to the west ward on the second floor, where a large company had assembled for the opening ceremony. The Duke of Marlborough read an address to their Majesties on behalf of the committee and governors of the institution. This stated that the committee of the King's Hospital Fund, anticipating great economies of management from a policy of amalgamation among special hospitals carrying on similar work in London, has urged the committees of the old Royal, National, and City Orthopædic Hospitals to amalgamate their funds, and therewith build a central institution. That building, which provided for 200 in-patients, principally children with malformations curable in the early stages, was the first realisation of the policy of concentration. Moreover, opposite the hospital a nurses' home had been provided and an out-patients' department, where last year 1,000 cases were treated, and where they had now installed a special orthopædic gymnasium. The cost of the buildings had amounted to about £75,000, of which £20,000 was still required. More than 400 patients were now waiting for admission.

His Majesty replied in terms of warm sympathy with the objects and principles of the new central institution, and in appreciation of the success which has attended the efforts of the authorities of the constitutent hospitals, whose work among crippled children the Queen has always watched with deep interest. His Majesty warmly approved of the centralisation principle, and commended to the charitable public this practical outcome of hospital concentration. After his Majesty's speech, prayers were said by the Bishop of London, and Lord Denbigh made a number of presentations to the King. Subsequently their Majesties, with Princess Victoria, visited some of the wards on the first floor.

The hospital has frontages on Great Portland Street and Bolsover Street. The buildings are designed to hold 200 beds in the wards and 13 on balconies, and a resident staff of 22. The nurses' home, accommodating 50 persons, and the out-patients' department are connected with the Hospital by a subway under Bolsover Street. Mr. Rowland Plumbe, F.R.I.B.A., is the architect.

The King has approved of the appointment of the following gentlemen to the consulting medical staff of King Edward VII. Sanatorium, Midhurst, Sussex: Dr. John Mitchell Bruce, M.D., F.R.C.P., and Dr. Bertrand Edward Dawson, M.D., F.R.C.P.

At the last meeting of the London County Council the following ladies and gentlemen were appointed to serve for three years on the medical staff for the inspection of school children in London: Messrs. O. L. Addison, M.B., F.R.C.S.; E. E. Argles, M.R.C.S., L.R.C.P.; J. M. Bernstein, M.B., M.R.C.P.; S. A. Boyd, M.S., F.R.C.S.; W. P. S. Branson, M.D., M.R.C.P.; G. Chaikin, M.R.C.S., L.R.C.P.; J. L. Dick, M.D., F.R.C.S.; J. G. Forbes, M.D., M.R.C.P.; C. F. Hadfield, M.D.; L. A. Hawkes, M.D.; C. W. Hogarth, M.R.C.S., L.R.C.P.; A. J. Jex-Blake, M.B., M.R.C.P.; D. W. C. Jones, M.B., M.R.C.P.; F. C. Lewis, M.D.; T. J. T. McHattie, M.B.; A. Mair, M.D.; G. Milne, M.D.; J. P. O'Hea, M.B., F.R.C.S.; F. W. Price, M.D., M.R.C.P.; G. E. C. Pritchard, M.D., M.R.C.P.; O. K. Williamson, M.D., M.R.C.P.; and A. S. Woodwark, M.B., M.R.C.P. Misses M. M. Burgess, M.D.; H. B. Hanson, M.D.; H. K. Whittingham, M.B.; and I. E. Woodward, M.B.

The Vacation Course in connection with the West London Postgraduate College, West London Hospital, Hammersmith Road, London, W., will commence on Monday, August 9.

### THE CHALFONT COLONY FOR EPILEPTICS.

The Sixteenth Annual Meeting of the Governors of the National Society for Epileptics, was held on Monday, July 19, at the London Offices, Denison House, Westminster. The chairman, Mr. Montefiore Micholls, said that during the past year a new home for 26 epileptic women was opened, and two homes for epileptic children (now completed) were commenced. Each of these three buildings cost about £3,500, and each was provided through the generosity of an individual donor—the Women's Home at the cost of Mr. Frederick Greene, the Boys' Home at that of the late Mr. C. A. Tate, and the Girls' Home at that of Mr. H. Woolcott Thompson. The Reports of the Executive Committee and Hon. Medical Staff for the year 1908 were received and adopted. From these it appears that the Colony has now accommodation for 224 adult patients, but since there are at least 40,000 epileptics in this country, of whom a considerable proportion would be suitable for treatment at Chalfont, the accommodation is clearly still quite inadequate.

